# Copy Number Variation in Patients with Disorders of Sex Development Due to 46,XY Gonadal Dysgenesis

**DOI:** 10.1371/journal.pone.0017793

**Published:** 2011-03-07

**Authors:** Stefan White, Thomas Ohnesorg, Amanda Notini, Kelly Roeszler, Jacqueline Hewitt, Hinda Daggag, Craig Smith, Erin Turbitt, Sonja Gustin, Jocelyn van den Bergen, Denise Miles, Patrick Western, Valerie Arboleda, Valerie Schumacher, Lavinia Gordon, Katrina Bell, Henrik Bengtsson, Terry Speed, John Hutson, Garry Warne, Vincent Harley, Peter Koopman, Eric Vilain, Andrew Sinclair

**Affiliations:** 1 Murdoch Childrens Research Institute, Royal Children's Hospital, Melbourne, Victoria, Australia; 2 Department of Paediatrics, The University of Melbourne, Melbourne, Victoria, Australia; 3 Department of Medical Genetics, University of California Los Angeles, Los Angeles, California, United States of America; 4 Pediatrics Department, Children's Hospital, Boston, Massachusetts, United States of America; 5 Walter and Eliza Hall Institute, Melbourne, Victoria, Australia; 6 Prince Henry's Institute of Medical Research, Melbourne, Victoria, Australia; 7 Institute for Molecular Bioscience, The University of Queensland, Brisbane, Queensland, Australia; Temasek Life Sciences Laboratory, Singapore

## Abstract

Disorders of sex development (DSD), ranging in severity from mild genital abnormalities to complete sex reversal, represent a major concern for patients and their families. DSD are often due to disruption of the genetic programs that regulate gonad development. Although some genes have been identified in these developmental pathways, the causative mutations have not been identified in more than 50% 46,XY DSD cases. We used the Affymetrix Genome-Wide Human SNP Array 6.0 to analyse copy number variation in 23 individuals with unexplained 46,XY DSD due to gonadal dysgenesis (GD). Here we describe three discrete changes in copy number that are the likely cause of the GD. Firstly, we identified a large duplication on the X chromosome that included *DAX1 (NR0B1)*. Secondly, we identified a rearrangement that appears to affect a novel gonad-specific regulatory region in a known testis gene, *SOX9*. Surprisingly this patient lacked any signs of campomelic dysplasia, suggesting that the deletion affected expression of SOX9 only in the gonad. Functional analysis of potential SRY binding sites within this deleted region identified five putative enhancers, suggesting that sequences additional to the known SRY-binding TES enhancer influence human testis-specific *SOX9* expression. Thirdly, we identified a small deletion immediately downstream of *GATA4*, supporting a role for *GATA4* in gonad development in humans. These CNV analyses give new insights into the pathways involved in human gonad development and dysfunction, and suggest that rearrangements of non-coding sequences disturbing gene regulation may account for significant proportion of DSD cases.

## Introduction

A defining point during embryogenesis is the commitment to develop as male or female. In males this is initiated by the Y-linked *SRY* gene, which leads to testis development. Ovarian development occurs in the absence of the Y-linked *SRY* gene, and ultimately results in a female phenotype. These developmental pathways involve complex networks of genes, the precise regulation of which is vital for the correct development of the gonads and associated anatomical structures [1].

Disruption of these networks can lead to disorders of sex development (DSD), which are congenital conditions with atypical development of the chromosomal, gonadal or anatomical sex [2]. DSD can be divided into three etiological subclasses, namely syndromic, disorders of androgen action, and gonadal dysgenesis [3]. The focus here is on individuals with 46,XY DSD due to gonadal dysgenesis (hereafter referred to as 46,XY GD). Individuals with 46,XY complete gonadal dysgenesis (CGD) are phenotypically female, have completely undeveloped streak gonads, and are often not diagnosed until puberty when secondary sexual characteristics fail to develop. Mutations in *SRY* and *SOX9* account for approximately 20% of 46,XY CGD patients [4]. Causative mutations affecting several other genes have been identified [5], including *DAX1 (NR0B1)* [6] *, SF1 (NR5A1)* [7], *WNT4* [8] *, DHH* [9] and *MAP3K1* [10]. Mutations in these genes are responsible for fewer than 30% of cases [11] and little is known about the underlying genetic basis in the remaining 50% of patients.

Individuals with 46,XY partial gonadal dysgenesis (PGD) and ovotesticular DSD have a phenotype that can range from a mildly undervirilised male through ambiguous genitalia to a slightly virilised female phenotype, depending on the amount of residual testicular function present. The diagnosis is therefore usually made at birth of the child with anomalous genitalia. In 46,XY ovarian DSD the phenotype is entirely female, including presence of female gonads. A diagnosis may be made due to presentation with early ovarian failure.

There have been intensive efforts in the last decade to identify novel genes involved in gonad differentiation, using a range of animal models [12], [13]. Although a large number of candidate genes have been identified, few mutations have been identified in these genes in human DSD patients. Even less is known about the gonad-specific regulation of these genes, although recent work has identified one gonad-specific enhancer of SOX9 in the mouse[14]. It is currently unclear whether unexplained cases of DSD are due to mutations in novel gonadal genes or mutations in the regulatory regions of known gonadal genes.

The genetic basis of several diseases has been elucidated by rare cases involving large, cytogenetically visible deletions or translocations that identified specific chromosomal regions for further analysis. For example, the region of the Y chromosome carrying the *SRY* gene was pinpointed by examining 46,XX testicular DSD patients with translocations of Y chromosome material to the X chromosome and 46,XY CGD patients who had deletions of the Y chromosome [15].

Copy number variation (CNV) is a term used to describe rearrangements of the genome such as deletions and duplications that result in an increase or decrease in the effective copy DNA number. The development of microarrays has allowed copy number analysis of the genome at a much finer resolution than was possible by cytogenetic analysis. This approach has been used to identify new disease genes in a range of disorders [16], [17]. This methodology has been applied to a few isolated DSD cases [18], [19], and has recently been used for studying diverse cohorts of DSD patients[20], [21]. Here we have used the Affymetrix Genome-Wide Human SNP Array 6.0 to identify copy number variants in 23 patients with DSD due to 46,XY gonadal dysgenesis.

## Materials and Methods

### Patient Information

Twenty-three unrelated patients were diagnosed as having 46,XY GD. All patients with a diagnosis of XY complete gonadal dysgenesis met the clinical criteria for this diagnosis, including female external genitalia associated with a 46, XY karyotype, the presence of Mullerian structures suggesting the lack of functional testicular tissue, and high levels of gonadotropins suggesting a primary gonadal failure caused by gonadal dysgenesis. Additional clinical features, when present, (such as adrenal hypoplasia), are indicated in Table 1.

**Table 1:** 46,XY GD cases studied.

Case #	Diagnosis	Other clinical features	Best candidate CNV (size/type)	Candidate gene(s)
1	46,XY ovotesticular DSD			
2	46,XY partial gonadal dysgenesis			
3	46,XY complete gonadal dysgenesis			
4	46,XY complete gonadal dysgenesis			
5	46,XY partial gonadal dysgenesis			
6	46,XY gonadal dysgenesis			
7	46,XY partial gonadal dysgenesis			
8	46,XY ovarian DSD			
9	46,XY complete gonadal dysgenesis			
10	46,XY complete gonadal dysgenesis	Cleft palate, short stature	Chr17:66200578-67393626 (1.193 Mb deletion)	*SOX9*
11	46,XY complete gonadal dysgenesis			
12	46,XY complete gonadal dysgenesis		Chr10:12382107-12770026 (388 kb duplication)	*CAMK1D*
13	46,XY complete gonadal dysgenesis		ChrX:30131772- 30902339 (771 kb duplication)	*DAX1* (*NR0B1*)
14	46,XY complete gonadal dysgenesis	Adrenal Hypoplasia Congenita	Chr8:11659702-11694481 (35 kb deletion)	*GATA4* (upstream of deletion)
15	46,XY complete gonadal dysgenesis		Chr13:42568370-42610053 (42 kb duplication)	*DNAJC15*
16	46,XY complete gonadal dysgenesis	Galactosemia		
17	46,XY complete gonadal dysgenesis			
18	46,XY complete gonadal dysgenesis	Short stature		
19	46,XY complete gonadal dysgenesis			
20	46,XY complete gonadal dysgenesis	Amelia		
21	46,XY complete gonadal dysgenesis	Adrenal Hypoplasia Congenita		
22	46,XY complete gonadal dysgenesis	IMAGE syndrome		
23	46,XY complete gonadal dysgenesis			

No mutation was detected in the *SRY* coding sequence in any of the 23 patients. For cases 14 and 21 (diagnosed with congenital adrenal hypoplasia) the *DAX1* and *SF1* genes had also been checked, and no mutation was identified. Genomic DNA from cases 1–8 was isolated from lymphoblastoid cell lines using standard methods. Genomic DNA from cases 9–23 was isolated from lymphocytes using standard methods. This study was approved by the Human Research Ethics Committee of the Royal Children's Hospital Melbourne, Australia (# 22073D).

### Affymetrix Microarray Analysis

All 23 DNA samples were hybridised onto the Affymetrix Genome-Wide Human SNP Array 6.0 microarrays. These arrays are composed of ∼1.8 million markers, evenly targeted at single nucleotide polymorphisms (SNPs) and other genomic regions allowing the identification of copy number variants. Microarray hybridisations were performed at the Australian Genome Research Facility (Melbourne, Australia) following manufacturer's instructions.

Data were analysed using an early-access version of the CRMAv2 total copy number (CN) method [22]. More precisely, crosstalk between alleles in (SNPs) was controlled for and global offset was removed from both SNPs and CN probes. The non-polymorphic SNP signals were estimated as the median probe-pair sum across replicated allele probe pairs (ignoring strand information). PCR fragment-length effects in the non-polymorphic SNP and CN probe signals were normalized. Total CNs were calculated as the ratio of the non-polymorphic signals relative to the robust average of all hybridizations. Chromosomal aberrations were identified from log2 CN ratios using the Gain and Loss Analysis of DNA (GLAD) 22. Duplications and deletions were called using log2-thresholds of +0.3 and −0.3, respectively, containing a minimum of 10 consecutive probes. The above analysis was conducted using aroma.affymetrix [23].

### Multiplex Ligation-dependent Probe Amplification (MLPA)

CNVs identified by microarray analysis were validated by Multiplex Ligation-dependent Probe Amplification (MLPA). Probes were designed according to previously described criteria [24]. Oligonucleotides were ordered from Sigma Genosys (Australia), and were desalted without further purification. The right hand oligonucleotide of each pair was 5′ phosphorylated to allow ligation to occur. Probe mixes were prepared by combining each oligonucleotide so that all were present at a final concentration of 4 fmol/µl in TE^−4^. MLPA reagents were purchased from Fisher Biotec (Australia). MLPA reactions and data analysis were performed as previously described [25], [26].

### DNA Sequencing

All primers used in sequencing were purchased from Sigma Genosys (Australia). Sequencing was conducted at either the Brisbane node of the Australian Genome Research Facility, or at the Department of Pathology, University of Melbourne.

### Putative SOX9 Enhancer

Putative SOX9 and SRY binding sites were identified using the HMR Conserved Transcription Factor Binding Sites track on the UCSC Genome Browser (hg18) with the default settings. PCR primers were designed to amplify seven conserved genomic regions (∼0.5–1.5 kb in size) containing putative SOX9 and SRY binding sites (table S1). The PCR fragments were cloned into pGL4.10 minSOX9 (SOX9 minimal promoter [27] driving the expression of firefly luciferase) upstream of the promoter.

### Dual Luciferase Assays

Luciferase assays were performed in a recently developed human cell line that expresses several markers of Sertoli cells [28], e.g. SOX9, GATA4 and AMH, with10^5^ cells plated in 24-well plates. After 24 hours, the cells were transfected with the relevant combination of plasmids using Lipofectamine 2000 (Invitrogen) according to the manufacturer's instructions. Briefly, cells in each well received 500 ng of luciferase reporter construct (pGL4-minSOX9 with or without putative enhancer fragments), 200 ng of transcription factor expression plasmid (pcDNA-SF1, SOX9, SRY or empty pcDNA), and 50 ng of pRL-TK as transfection control. After 48 hours, cells were harvested and luciferase activity was measured using the Dual Luciferase Assay Kit (Promega) in a TD-20/20 Luminometer (Turner Designs). Values were normalized for transfection efficiency with respect to the effects of the expression constructs on the empty pGL4-minSOX9 reporter plasmid. Relative light units shown represent mean values ±SEM obtained from at least four independent experiments performed in duplicate.

### RNA Isolation and Amplification

Gonads were dissected from E12.5-E15.5 *Oct4-GFP* mouse embryos, dissociated with trypsin and FACS sorted into germ and somatic fractions. Total RNA was isolated and amplified as previously described [29].

### Reverse Transcription and Real-Time PCR Analysis

Amplified RNA was reverse transcribed using the Transcriptor High Fidelity cDNA synthesis kit (Roche, Mannheim, Germany). Briefly, 100 ng amplified RNA was mixed with 2 µl of Random Hexamer Primers, denatured at 65°C for 10 minutes and immediately cooled on ice. Samples were reverse transcribed following addition of 4 µl of 5x Transcriptor High Fidelity Reverse Transcriptase Reaction Buffer, 0.5 µl of Protector RNase Inhibitor, 2 µl of Deoxynucleotide Mix, 1 µl of DTT and 1 µl of Transcriptor High Fidelity Reverse Transcriptase at 50°C for 30 minutes and inactivated at 85°C for 5 minutes.

Real-time PCR was performed in triplicate using 1 ng cDNA in each 10 µl reaction, using the mouse Universal Probe Library system (Roche), LightCycler 480 Probe Master mix (Roche) and a LightCycler 480 instrument (Roche). All primers were designed using the UPL Assay Design Centre (https://www.roche-applied-science.com). Relative expression was determined using the comparative C_T_ method (ΔΔC_T_), with samples normalised against *Sdha* and expressed relative to the sample showing the lowest level of expression for each experiment. In addition, the efficiency of each primer/probe combination was determined using standard curves.

## Results

### Microarray analysis

Twenty-three unrelated patients were diagnosed as having 46,XY GD. No mutation was detected in the *SRY* coding sequence in any of the patients. We screened the genomic DNA of these patients using the Affymetrix Genome-Wide Human SNP Array 6.0. A stringent threshold of at least 10 consecutive probes was set for a CNV to be called. Using this criterion 1,498 high-confidence CNV regions were identified, with no difference in the average number or size of CNVs detected between DNA isolated from cell lines or lymphocytes (Table 2).

**Table 2:** CNV analysis using the Affymetrix 6.0 array.

	Cases 1–8 (derived from cell lines)	Cases 9–23 (derived from lymphocytes)
Number of samples	8	15
CNVs/genome	64	66
Min/max/median CNV size (kb)	0.4/1577/20	0.3/1778/19
>50% overlap with known CNVs	92%	90%

Of the CNVs detected, 91% overlapped for at least 50% with previously reported CNVs in the database of genomic variants (DGV). To prioritise regions for further analysis we focussed on rearrangements that a) covered or were within 500 kb of genes known to be involved in gonad development; or b) affected the coding region of RefSeq genes and were not listed in the DGV.

### Rearrangements affecting known gonadal genes

**Figure 1:**
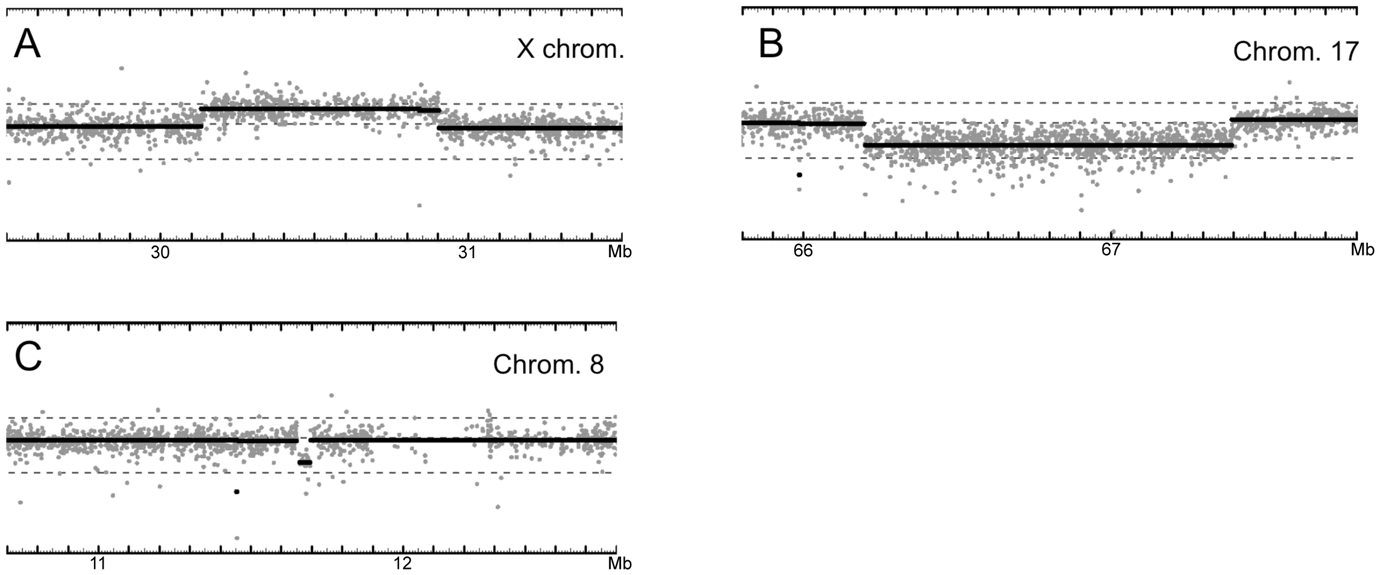
CNV analysis of three 46,XY GD cases using the AROMA algorithm. Data are plotted along each chromosome, with each point represents the copy number estimate of an individual probe. The horizontal solid black line denotes the predicted copy number of the genomic region. For each panel coverage of 2 Mb is shown, with numbers on the horizontal axis corresponding to the March 2006 human reference sequence (hg18). A) Duplication of ∼708 kb on the X-chromosome in case 13. B) Deletion of 1.193 Mb on chromosome 17 in case 10. C) Deletion of 35 kb on chromosome 8 in case 14.

Three cases had rearrangements that affected genes known to play a role in sex determination or gonad development (for all CNVs identified in these cases see table S2). Firstly, a 708 kb duplication on the X chromosome was identified in case 13 (Figure 1a). This 46,XY DSD patient was diagnosed with complete gonadal dysgenesis and no other clinical features were reported. One of the seven genes contained within the duplicated region was *DAX1 (NR0B1),* which has been previously been shown to be duplicated in 46,XY complete gonadal dysgenesis.

**Figure 2:**
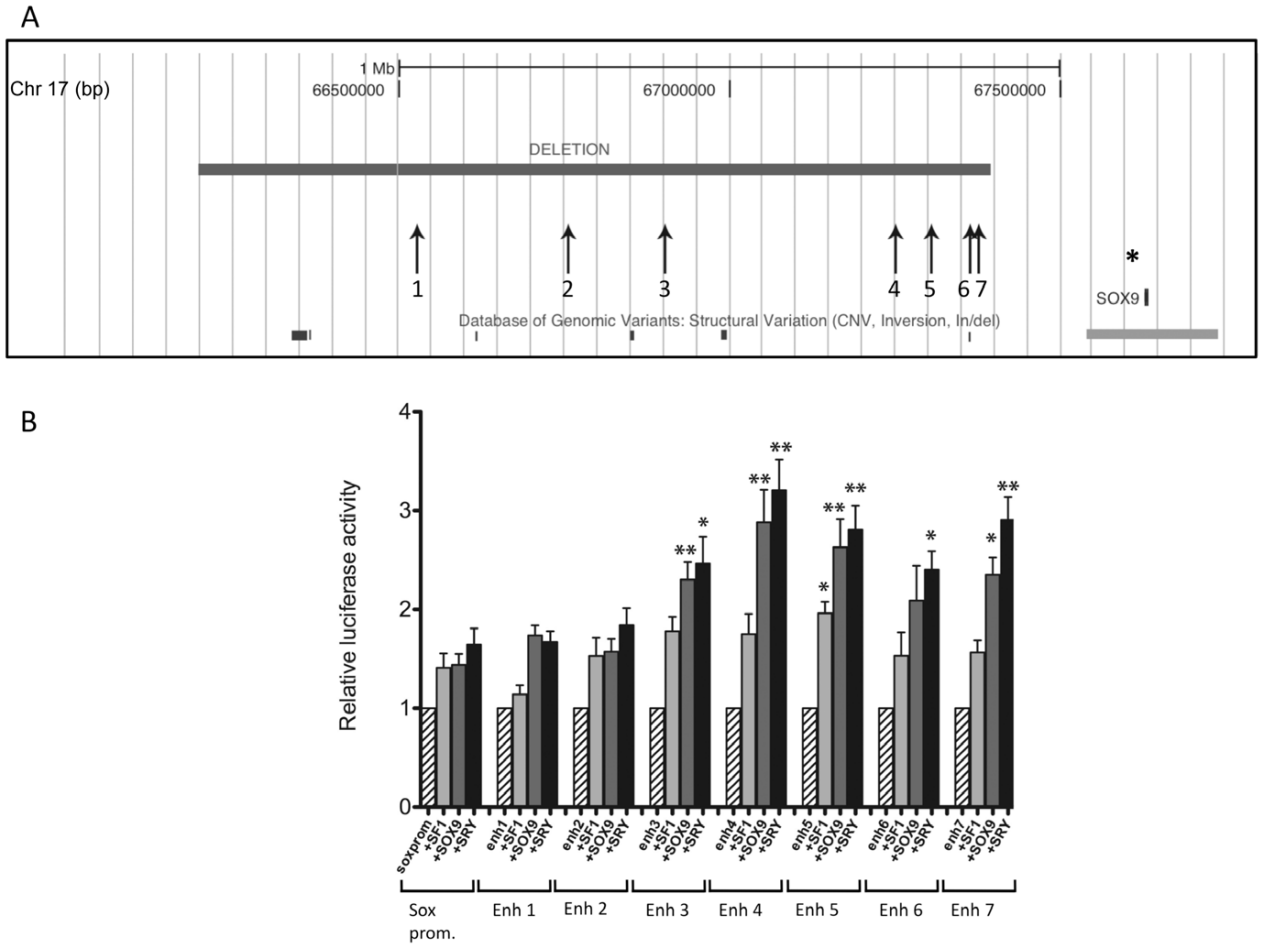
Deletion (1.193 Mb) on chromosome 17, upstream of *SOX9* in a patient with 46,XY GD. a) The location and extent of the 1.193 Mb deletion on chromosome 17, upstream of *SOX9* identified in case 10. The numbers at the top of the figure correspond to nucleotide position based on the March 2006 human reference sequence (hg18). Also shown are structural variants within this region that are listed in the database of genomic variants. (http://projects.tcag.ca/variation/). The numbered arrows indicate the positions of the seven potential gonad specific regulatory elements (enh1–7) that were cloned into reporter constructs. The position of the orthologous sequence corresponding to the mouse TESCO sequence is indicated by an asterisk (*). b). Reporter construct analysis of SOX9 regulatory regions. Effect of selected transcription factors on luciferase activity driven by putative gonad regulatory regions (enh1–7) inserted upstream of the minimal SOX9 promoter (sox prom). Results are given as relative activation of the reporter by the expression constructs (SF1, SOX9, SRY) compared with the empty vector (pcDNA3). Data represent mean values ±SEM obtained from at least four independent experiments. Statistical analysis was performed with a 2-tailed t-test. ** p<0.005; * p<0.05.

Secondly, a deletion of 1.193 Mb approximately 300 kb upstream of the *SOX9* gene was identified in case 10 (Figure 1b). This 46,XY individual had been diagnosed with complete gonadal dysgenesis. The only other clinical features reported were a cleft palate and height at the 3^rd^ centile. This is very unusual as rearrangements and mutations affecting *SOX9* normally result in patients with campomelic dysplasia (deformity of chondrogenesis) and in some 46,XY cases, gondal dysgenesis. *SOX9* is known to play a critical role in testis development, and other rearrangements upstream of this gene have been described in 46,XY DSD (or CGD) patients. The deletion described here did not cover the orthologous sequence of the recently identified TESCO element in mouse, which was proposed to act as an enhancer in regulating SOX9 expression in the gonad [14]. The orthologous human TESCO region was sequenced in the index patient, and no mutations or polymorphisms were identified (data not shown). The gonadal dysgenesis observed in this patient suggests that one or more testis-specific regulatory elements of SOX9 are contained within the 1.2 Mb deletion. In an attempt to identify such regions we performed a bioinformatic analysis of the 1.2 Mb region, searching for potential SRY/SOX9 binding sites that were conserved in human, mouse and rat. In total we identified seven such regions, hereafter referred to as enh1–7 (Figure 2a). Each of these regions was PCR amplified and cloned into a pGL4 vector containing the human SOX9 minimal promoter. Following transfection into a human Sertoli cell line, Addition of SF1, SRY or SOX9 led to a statistically higher level of enhancer activity for enh3, 4, 5, 6 and 7 for at least one of the transcription factors, with enh5 affected by all three (Figure 2b).

**Figure 3:**
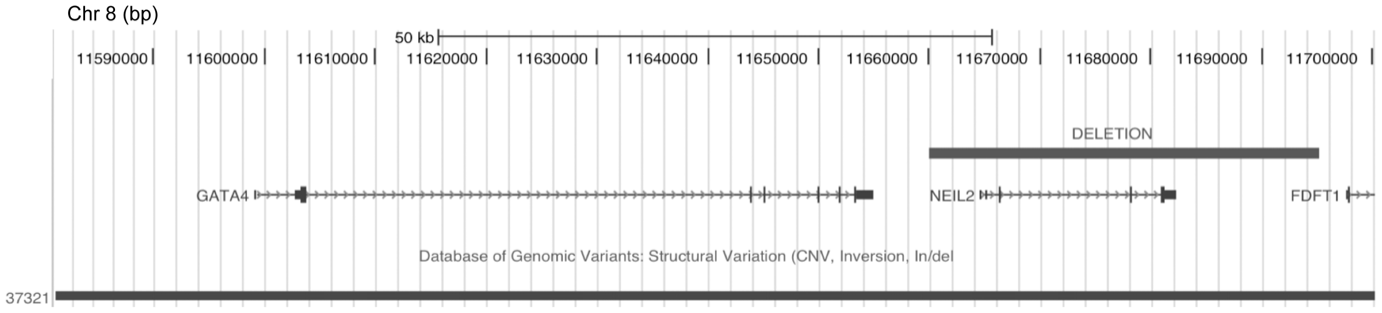
Deletion (35 kb) on chromosome 8, downstream of *GATA4* in a patient with 46,XY GD. The minimum size of the deletion as defined by array analysis is shown. The numbers at the top of the figure correspond to nucleotide position based on the March 2006 human reference sequence (hg18). Also shown is an inversion covering this region that is listed in the database of genomic variants (http://projects.tcag.ca/variation/).

Thirdly, a 35 kb deletion including *NEIL2* was identified in case 14 (Figure 1c and Figure 3). Although there is no known role for *NEIL2* in sex determination or differentiation, the deletion was immediately downstream of *GATA4*, a gene previously implicated in gonad development in the mouse. Low microarray probe density around the deletion breakpoint made it unclear whether the *GATA4* 3′UTR was included in the deletion. Several MLPA probes were designed in the intervening space, and analysis showed that the deletion did not extend into *GATA4* 3′ UTR sequence (data not shown). The deletion was not present in the father or unaffected sister (DNA from the mother was not available for analysis), and sequence analysis did not reveal any variants in either *NEIL2* or *GATA4* in the index patient.

### Rearrangements affecting candidate gonadal genes

**Figure 4:**
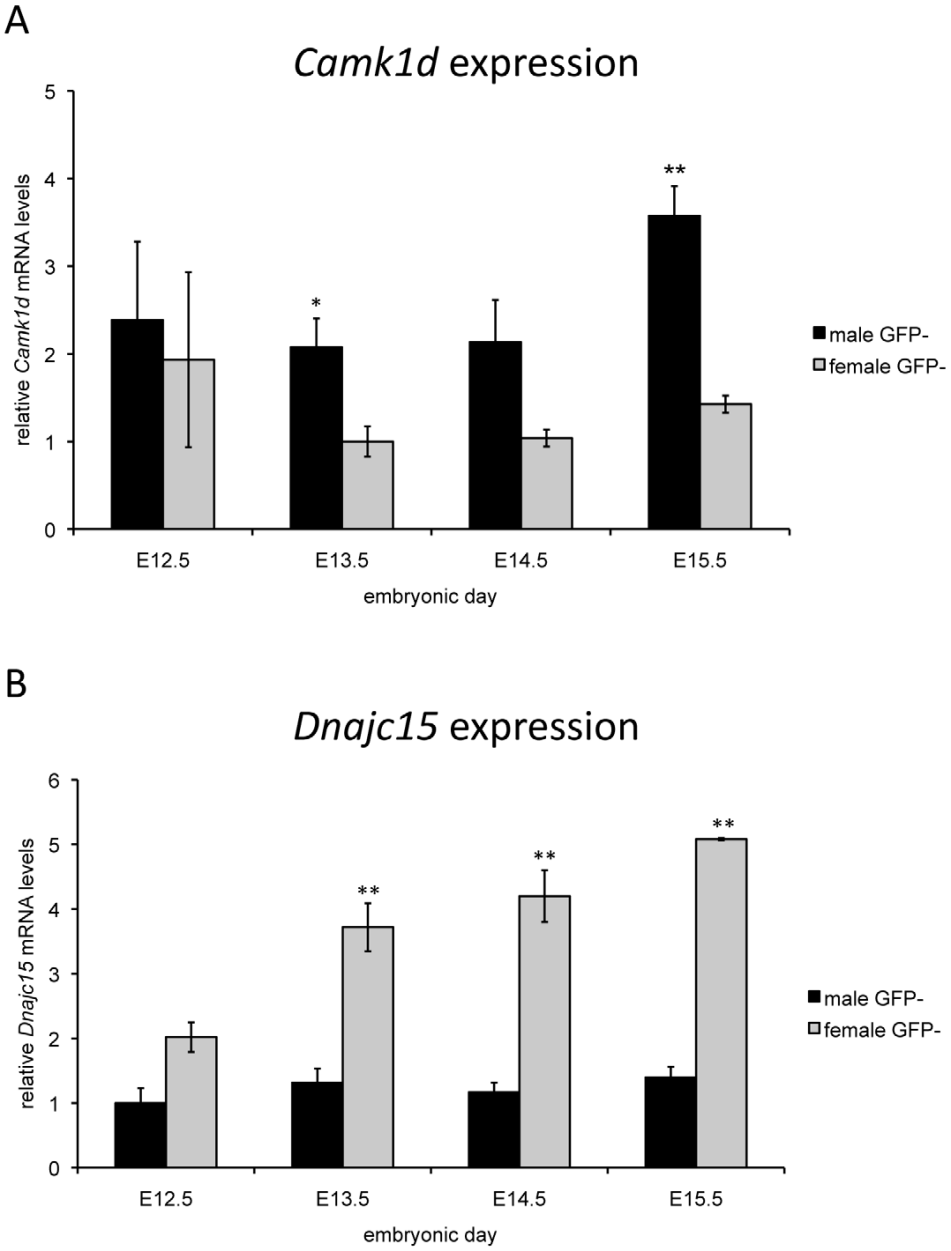
Expression analysis of *Camk1d* and *Dnajc15* in developing mouse gonads. Expression analysis was performed on cDNA from sorted GFP- (somatic) cells derived from male and female mouse gonads, embryonic days 12.5–15.5. The data is normalised such that the lowest expression is 1.0, and represent mean values ±SEM from three independent experiments. Shown here are the expression patterns for a) *Camk1d* and b) *Dnajc15*. Comparisons between male and female expression for *Camk1d* and *Dnajc15* were performed with a 2-tailed t-test. ** p<0.005; * p<0.05.

Several previously unreported CNVs were identified that affected the coding region of genes not currently associated with gonad development. The genes affected were *GPR83, ACBD7, CAMK1D, OLAH*, *NEIL2* and *DNAJC15* (table S3). To assess the potential of these genes to be involved in DSD, expression analysis of the orthologous mouse genes was performed on cDNA from purified somatic cells isolated from male and female mouse gonads at the time of sex differentiation. Two genes (*Dnajc15* and *Camk1d*) showed sexually dimorphic expression in the somatic cells (Figure 4). *Dnajc15* was expressed more highly in the female gonad than the male (p<0.005 at E13.5, E14.5, E15.5). Conversely, *Camk1d* expression was significantly higher in the developing male gonad when compared to the female gonad (p<0.05 at E13.5, p<0.005 at E15.5).

## Discussion

We have used the Affymetrix Genome-Wide Human SNP Array 6.0 to identify deletions and duplications in a cohort of 23 patients with 46,XY GD. A relatively stringent threshold of 10 consecutive probes was set before a CNV was considered genuine. However, despite this stringent criterion over 65 CNVs per sample were detected by the microarray. Discriminating causative CNVs from benign polymorphisms was complicated by the incomplete knowledge of polymorphic CNVs [30]. When comparing our findings with those reported in the database of genomic variants [31], over 90% overlapped with previously identified rearrangements for at least half the affected region. A confounding issue associated with any mutation screening study, but particularly relevant for DSD, is parent of origin effect. As gonad development in males and females involves distinct genes and pathways, a given variant might disrupt one gonad specific pathway, leading to DSD, whilst having no effect on the development of the other gonad type. It is therefore not possible to completely exclude CNVs that have been inherited (particularly from the parent with the opposite sex chromosome composition or that have previously been identified in apparently healthy individuals. Therefore, in addition to previously undescribed CNVs affecting RefSeq genes we have also chosen to examine rearrangements overlapping or in proximity to genes known to be involved in gonad development, irrespective of whether or not they overlap with previously reported CNVs.

### *DAX1 (NROB1)*

The first rearrangement was a 708 kb duplication containing *DAX1* in case 13. Other 46,XY GD patients have been described with duplications overlapping the same region of the X chromosome [19], [32]. *DAX1* shows sexually dimorphic gonadal expression, being downregulated in the developing testis [33]. Studies in mouse models support *Dax1* as having both pro-testis [34], [35] and anti-testis functions [33]. These studies indicate that a critical level of *Dax1* is required for proper testis cord formation – levels above or below this level interfere with the early stages of testicular differentiation (e.g, a duplication interferes with SRY function, while a loss of function retards early cord organisation on a susceptible genetic background). As testis development proceeds, DAX1 is down-regulated, that is it antagonises the SF1/WT1/SOX9 activation of AMH. Hence, DAX1 has dynamic effects on the developing testis that changes over time.[36]

### *SOX9*

The second rearrangement affecting a known testis gene was a 1.2 Mb deletion, approximately 300 kb upstream of *SOX9* in case 10. Several studies have demonstrated that correct expression of *SOX9* is critical for appropriate development of chondrogenic tissue [37] and testes [38]. A large duplication encompassing *SOX9* was reported in a single, mosaic case of 46,XX testicular DSD [39]. This rearrangement presumably led to increased levels of SOX9 expression and testis development. Mutations within and outside of *SOX9* are known to cause the bone disorder campomelic dysplasia (CD), which in approximately 70% of cases is associated with 46,XY GD. Non-overlapping deletions and translocation breakpoints up to 1 Mb from the *SOX9* coding sequence have been identified in CD patients, with and without 46,XY GD), suggesting that there are multiple regulatory elements controlling SOX9 expression[40], [41], [42], [43], [44], [45], [46], [47], [48]. Case 10 is remarkable as it is the first reported case of a rearrangement affecting the *SOX9* locus in a 46,XY GD individual in the absence of campomelic or acampomelic dysplasia. The only clinical features reported other than gonadal dysgenesis were short height (3^rd^ centile) and a cleft palate. The deletion in case 10 does not overlap with the cis-regulatory element identified in a study of Pierre-Robin sequence patients [49], a disorder that presents with several clinical features including cleft palate. The close proximity (∼13 kb from) however, suggests that a position effect altering the function of this putative enhancer may underlie the cleft palate observed in case 10.

The lack of any bone pathology consistent with CD would suggest that the regulatory element(s) necessary for appropriate *SOX9* expression during skeletal development are present outside the deleted region in this patient. Furthermore, it suggests that mutations affecting *SOX9* regulation in the gonad may have been underestimated as a cause of gonadal dysgenesis in the absence of CD. However, we cannot exclude that the deletion has created a new regulatory element, or relocated an existing sequence that can function as a tissue-specific silencer. This would be analogous to a previously described insertion/deletion upstream of the *Sox9* locus in a XX sex reversed transgenic mouse [50]. In that instance it was originally thought that the deletion removed a gonad-specific suppressive element, but subsequent studies showed that the inserted material was capable of driving *Sox9* expression in the mouse gonad [51].

In case 10 the deletion upstream of SOX9 did not extend into the orthologous human sequence of a recently identified enhancer that has been proposed to regulate *Sox9* expression in the developing mouse gonad [14]. In mouse a 3.2 kb sequence (TES – testis-specific enhancer) ∼10 kb upstream of *Sox9* was defined, with 1.4 kb of this (TESCO – TES core element) as the critical region. Functional studies showed that the TESCO sequence was capable of acting as an enhancer in an Sry- and Sf1-dependent manner, consistent with Sox9 being a direct downstream target of Sry. Sf1 and Sry were shown to be required for enhancer activity, as mutating their binding sites within the TES sequence eliminated enhancer activity. However, it has not yet been demonstrated that deletion of the TESCO region is sufficient by itself to cause male to female sex reversal. Indeed, a recent study of 66 46,XY gonadal dysgenesis patients did not identify any mutations in the genomic region orthologous to TESCO [52]. We sequenced this region in case 10, and did not identify any sequence variants. As no point mutations have been identified in the TESCO region of 46,XY patients, and loss-of-function rearrangements outside this region have been identified in other 46,XY GD patients, regulatory elements other than or in addition to TESCO are likely to be involved in gonad-specific *SOX9* expression in humans.

Common bioinformatic approaches to identify potential regulatory elements include examining sequences that show significant interspecies conservation, or sequences containing consensus binding site motifs for transcription factors. Previous studies have used these approaches to identify putative *Sox9* enhancers in mice [53], [54]. We selected seven human DNA sequences within the region deleted in case 10 that are conserved between human, mouse and rat and contain putative SRY/SOX9 binding sites. Co-transfecting reporter constructs containing each of these sequences together with SRY, SF1 or SOX9 into a human Sertoli cell line revealed that five of the seven regions showed significantly increased luciferase expression when compared to the SOX9 minimal promoter only. Enhancer 5 (enh5) was the only one to show a significant increase in luciferase expression for each of the transcription factors, and was the only one to respond to SF1. Enh6 responded only to SRY, whereas each of enh3, enh4 and enh7 responded to both SOX9 and SRY. These DNA sequences are the first putative SOX9 gonad enhancers identified in humans, and are targets for mutation screening in a larger cohort of 46,XY DSD GD patients.

### *NEIL2* and *GATA4*

We identified a 35 kb deletion that completely removed *NEIL2* in case 14. Although there is no evidence supporting a role for *NEIL2* in testis determination, the deletion is immediately 3′ of the neighbouring *GATA4* gene. In mice, mutations of *Gata4* that interfere with Gata4-Fog2 interaction lead to gonadal abnormalities in specific strains of XY mice [55]. It has been shown that Gata4 also acts synergistically with Wt1 in activating both the *Sry* and *Amh* promoters.[56] Amh (anti-Müllerian hormone, also known as Müllerian inhibiting substance) is responsible for the regression of the Müllerian structures that would otherwise form the female internal reproductive tract. Mutations affecting the coding sequence of *GATA4* in humans are associated with cardiac malformations, with no mutations as yet associated with gonadal dysgenesis. As the rearrangement we have identified does not affect *GATA4* coding sequence, we would not necessarily expect cardiac malformations as a consequence of the deletion. It is possible that a regulatory element controlling *GATA4* expression in the developing gonad is either removed or relocated by the deletion. This is analogous to the situation we recently described for rearrangements nearby the SOX3 coding sequence in 46,XX males which affected gene regulation [57].

While this manuscript was under revision there was a report describing a family where a *GATA4* missense mutation segregated with congenital heart disease and 46,XY DSD[58]. There were three affected 46,XY males with DSD. All three patients had inguinal gonads (in one individual the gonads were removed surgically as they were dysgenic) and two had hypospadias. In addition one of the males had a systolic murmur without any atrial septal defect. Two 46,XX females within the family had congenital heart conditions, although there was no sign of gonadal dysfunction.

The authors proposed that the DSD observed in 46,XY carriers of the *GATA4* mutation was due to a loss-of-function effect on GATA4 in the developing gonad, interfering with its ability to interact with FOG2 and disturbing activation of the *AMH* promoter together with NR5A1. This finding further strengthens the case for the deletion we observed downstream of *GATA4* in case 14 as being causative for DSD.

### Other CNVs

We identified several other previously unreported CNVs that affected the coding region of genes. To assess the potential role of any of these genes in gonadal development we examined their expression levels in FACS sorted somatic cells from E12.5 to E15.5 mouse gonads. The advantage of using a purified cell population for expression analysis is that testis determining genes are expected to be enriched specifically in the somatic component [1]. Two genes (*Dnajc15* and *Camk1d*) showed sexually dimorphic expression in somatic cells. It is interesting to note that the two genes show opposite patterns of expression, as *Camk1d* is higher in the male gonad vs the female whereas *Dnajc15* is higher in the female. Although this is apparently contradictory in patients with similar clinical features, it may be that the genes have opposite roles in testis development *i.e.* one has a positive effect and the other has an inhibitory function.

*Dnajc15* has been suggested to be involved in pronephros development in Xenopus embryos [59], and *Camk1d* has been associated with type 2 diabetes [60]. Further analysis needs to conducted on these genes to define their potential role in gonad development.

Our analysis of 23 patients identified a large number of previously unidentified CNVs outside coding sequence. Unfortunately it is currently impractical to assay the effects of each of these in mouse models. It is possible that there may be as yet unidentified genes or non-coding RNAs within these regions. Further analysis of potential regulatory elements looking at markers such as histone modifications [61] or DNaseI hypersensitive sites [62], [63] in chromatin are powerful approaches to prioritise regions for more detailed analysis.

There are a number of possible explanations for the remaining patients in whom no causative mutations were identified. Point mutations in the known sex determining genes may account for some cases, although previous studies suggest that these will explain less than 20% of all cases of 46,XY GD [11]. Smaller rearrangements, within these or other genes that are beneath the resolution of the microarray will have been missed in this study. In addition, there may be point mutations in other, as yet unidentified sex determining genes, and it is likely that mutations affecting regulatory elements in non-coding regions of the genome are responsible for a number of cases.

In conclusion, we have identified a number of potentially causative genomic rearrangements in patients with 46,XY GD. These include a duplication of *DAX1 (NROB1)* and a deletion upstream of *SOX9* which potentially contain novel gonad-specific gene regulatory elements. In addition, we found a deletion in close proximity to *GATA4*. This rearrangement is the first evidence in humans suggesting that mutations affecting GATA4 expression may be involved in 46,XY GD. Our findings suggest that rearrangements of non-coding sequences that disturb gene regulation may account for significant proportion of DSD cases, suggesting that new strategies will be required for increasing diagnostic yields. Characterising the regulatory elements responsible for the correct spatial and temporal expression of these genes will be necessary to obtain a true picture of the gene networks responsible for gonad differentiation and development.

## Supporting Information

Table S1 SOX9 regulatory region PCR primers. The PCR primers used to amplify candidate SOX9 regulatory regions. Chromosomal locations are based on the March 2006 human reference sequence (hg18).
(DOC)

Table S2 46,XY GD cases. All CNVs detected in cases 10, 13, 14. Chromosomal locations are based on the March 2006 human reference sequence (hg18).
(XLS)

Table S3 CNV analysis using the Affymetrix 6.0 array. Gene-containing CNVs detected in this study that were not found in the database of genomic variants. Chromosomal locations are based on the March 2006 human reference sequence (hg18).
(DOC)
