# Supplementary material for: Copy Number Variation in Patients with Disorders of Sex Development Due to 46,XY Gonadal Dysgenesis
Source: PLoS One. 2011 Mar 7;6(3):e17793. doi: 10.1371/journal.pone.0017793 (PMC3049794; doi:10.1371/journal.pone.0017793)
Supplement: Table S1 — SOX9 regulatory region PCR primers. The PCR primers used to amplify candidate SOX9 regulatory regions. Chromosomal locations are based on the March 2006 human reference sequence (hg18). (DOC) [file pone.0017793.s001.doc]

| Enhancer1-for  Enhancer1-rev | ATCGCTAGCGGTTGAGTCAAACTAAGGTACC  GTAGCTAGCATCAATTTCAGTCACTCATTCAC  Chr17: 66531234-66532525 |
| --- | --- |
| Enhancer2-for  Enhancer2-rev | ATATGCTAGCCTTTCCCTATCCCCAAGTCTG  ATATGCTAGCCCATTGTACTTTAAATGTTCTTCTCC  Chr17: 66759136-66760156 |
| Enhancer3-for  Enhancer3-rev | AATGCTAGCCTCCAATAAATCTACCGTGAGC  TTAGCTAGCCACCTATAGCTAACCTCAGCTAGAG  Chr17: 66906031-66907149 |
| Enhancer4-for  Enhancer4-rev | ATATGCTAGCGAAGGAGAAGATGACTTGGTCAC  ATATGCTAGCATGCATGCTGTTTGAATTCAG  Chr17: 67251184-67251726 |
| Enhancer5-for  Enhancer5-rev | ATATGCTAGCCTTCTTCCCAAGTCTGTCTCTG  TCCTAGGCACATCTAGAAATGG  Chr17: 67308793-67309512 |
| Enhancer6-for  Enhancer6-rev | ATATGCTAGCACTTAAAGCCCAGACTTCACC  ATATGCTAGCACTTCTACTAAGCCACTCAGAAGC  Chr17: 67362414-67363560 |
| Enhancer7-for  Enhancer7-rev | ATATGCTAGCGTGCATTTTACTTGCTCTTGC  ATATGCTAGCCCTTTTACGACTCTCACCTGG  Chr17: 67373263-67374286 |
