# Supplementary material for: Copy Number Variation in Patients with Disorders of Sex Development Due to 46,XY Gonadal Dysgenesis
Source: PLoS One. 2011 Mar 7;6(3):e17793. doi: 10.1371/journal.pone.0017793 (PMC3049794; doi:10.1371/journal.pone.0017793)
Supplement: Table S3 — CNV analysis using the Affymetrix 6.0 array. Gene-containing CNVs detected in this study that were not found in the database of genomic variants. Chromosomal locations are based on the March 2006 human reference sequence (hg18). (DOC) [file pone.0017793.s003.doc]

| **Patient** | **Rearrangement** | **Locus** | **Size** | **Gene** | **Primary cell type for expression** |
| --- | --- | --- | --- | --- | --- |
| *3* | Whole gene deletion | Chr11:93697501-93798531 | *101 kb* | *Gpr83* | germ |
| *11* | Whole gene duplication | Chr10:15066053-15179639 | *114 kb* | *Olah* | germ |
| *11* | Whole gene duplication | Chr10:15066053-15179639 | *114 kb* | *Acbd7* | germ |
| *12* | Partial gene duplication | Chr10:12382107-12770026 | *388 kb* | *Camk1d* | somatic |
| *14* | Whole gene deletion | Chr8:11659702-11694481 | *35 kb* | *Neil2* | germ |
| *15* | Partial gene duplication | Chr13:42568370-42610053 | *42 kb* | *Dnajc15* | somatic |
